# Dynamics of Rotating Micropolar Fluid over a Stretch Surface: The Case of Linear and Quadratic Convection Significance in Thermal Management †

**DOI:** 10.3390/nano12183100

**Published:** 2022-09-07

**Authors:** Bagh Ali, N. Ameer Ahammad, Aziz Ullah Awan, Kamel Guedri, ElSayed M. Tag-ElDin, Sonia Majeed

**Affiliations:** 1 School of Mathematics and Statistics, Northwestern Polytechnical University, Xi’an 710072, China; 2 Faculty of Computer Science and Information Technology, Superior University, Lahore 54000, Pakistan; 3 Department of Mathematics, Faculty of Science, University of Tabuk, P.O. Box 741, Tabuk 71491, Saudi Arabia; 4 Department of Mathematics, University of the Punjab, Lahore 54590, Pakistan; 5 Mechanical Engineering Department, College of Engineering and Islamic Architecture, Umm Al-Qura University, P.O. Box 5555, Makkah 21955, Saudi Arabia; 6 Faculty of Engineering and Technology, Future University in Egypt, New Cairo 11835, Egypt

**Keywords:** linear and nonlinear convection, micro-polar fluid, MHD, rotating frame, thermal management

## Abstract

This article analyzes the significance of linear and quadratic convection on the dynamics of micropolar fluid due to a stretching surface in the presence of magnetic force and a rotational frame. Modern technological implementations have attracted researchers to inquire about non-Newtonian fluids, so the effect of linear and nonlinear convection conditions is accounted for in the dynamics of non-Newtonian fluid. The highly nonlinear governing equations are converted into a system of dimensionless ODEs by using suitable similarity transformations. The bvp4c technique is applied in MATLAB software to obtain a numerical solution. This investigation examines the behavior of various parameters with and without quadratic convection on the micro-rotation, velocity, and temperature profiles via graphical consequences. The velocity profile decreases with a higher input by magnetic and rotating parameters, and fluid velocity is more elevated in the nonlinear convection case. However, the temperature profile shows increasing behavior for these parameters and quadratic convection increases the velocity profile but has an opposite tendency for the temperature distribution. The micro-rotation distribution is augmented for higher magnetic inputs in linear convection but reduces against thermal buoyancy.

## 1. Introduction

The mixing and flowing of fluids are generated through density variation caused by differences in temperature within the fluid, known as natural convection. The fluid density near the heated surface is lower than that of the cold fluid. Gravity raises the hot fluid upward, causing the buoyant force to manifest. The convective flux problems of working liquid have various engineering implementations, such as food preservation, cryogenic devices, solar collectors, energy storage, and nuclear reactor technology. Nonlinear convection exists in the buoyancy force because of temperature fluctuations substantially affecting flux characteristics. Awan et al. [1] analyzed viscous liquid’s free convection flux along with mass fluxes and damped thermal effects. Mixed convection in ducts and pipe flux along strong magnetic fields is explored by Zikanov et al. [2]. Liaqat et al. [3] and Armaghani et al. [4] examined the mixed convection heat transportation of nanoliquids and hybrid nanoliquids subject to stretched surfaces.

The fluids class showing microscopic change induced by micro-rotation and the local formation of fluid molecules are known as microfluids. The notion of this type of fluid was firstly offered by Eringen et al. [5]. In recent years, the theory of micropolar fluids has gained more importance for its various valuable applications, such as polymeric suspensions, liquid crystals, biological structures, nuclear power plants, the flux of turbulent shear, and slurries. Galdi et al. [6] explored the uniqueness and existence of the solutions to the micropolar equations. Nazar et al. [7] inquired about the stagnation point flux of micropolar liquid towards an extending sheet. Yusuf et al. [8] illustrated the irreversible impact in the micropolar liquid film with an incline permeable substrate along slip effects. Bilal et al. [9] studied a parametric simulation of micropolar liquid experiencing heat radiation on a permeable extending surface. Many researchers have recently worked on non-Newtonian fluid subject to different circumstances [10,11,12].

The study of magnetohydrodynamics (MHD) in liquid flux gains importance in handling flux characteristics. Essentially, MHD is concerned with the magnetic properties of electrically conductive liquid. The effects of MHD on fluid flow have been observed in several real-world instances in chemistry, physics, and industry such as stream meters, crystal growth, pumps, power plants, controlled thermonuclear reactors, wire drawing, MHD generators, and oil recovery techniques. Experimental and theoretical analysis of magnetohydrodynamic pumps is explored by Jang et al. [13]. Ali et al. [14] examined the MHD heat and mass transmission on an expanding sheet in rotational nanoliquid with binary chemical reaction, swimming microorganisms, and non-Fourier heat flow. Chabani et al. [15] reported the MHD flux of hybrid nano liquid in different shapes. Nadeem et al. [16] illustrated models based on the study of inclined MHD of hybrid nanoliquid flux on nonlinear extending cylinders. Sajid et al. [17] analyzed the MHD revolving flux of viscous fluid on a shrinking sheet.

Recently, analyzing energy and fluid transport problems in a rotational frame has been widely studied in various mathematics models and turned into a fascinating phenomenon. It is due to their tremendous applications in the food industry, cosmic liquid dynamics, viscometry, and computer stockpiling devices. Since rotational flux is critical, researchers attempt to comprehend the underlying science. The foremost endeavor of this pathway was developed by Wang [18]. Dessie et al. [19] reported the influence of chemical reactions, thermal energy, and activation energy on MHD Maxwell liquid flux in a revolving frame. Nadeem et al. [20] performed a numerical analysis of water-based carbon nanotubes of micropolar liquid in a rotating frame. Spear et al. [21] explained the new insights in rotating frame relaxation at high field strength. Shahzad et al. [22] described the heat transmission analysis of MHD rotational flux of $Fe_{3}O_{4}$ by extending the surface.

This analysis investigates the significance of linear and nonlinear convection on the dynamics of micropolar fluid and heat transfer across a horizontal expanding surface. The current study has the following novelties:

(i) incorporate the micropolar fluid as host fluid,
(ii) analyze with and without quadratic convection on the dynamic of micropolar fluid, and
(iii) consider the significant effects of magnetohydrodynamics subject to a rotating frame of reference.

Since the authors pointed out that none of the cited studies above are focused on the elaborated problem, the bvp4c technique is applied, and differentiated outcomes for micro-rotation, velocity, temperature, skin friction, and Nusselt number are obtained.

### Research Questions

This analysis gives scientific answers to the following research questions:

(1) To explore the significance of material, magnetic, and rotating parameters on the dynamic of microplar fluid: the case of linear and quadratic convection.
(2) To observe the influence of Lorentz and Coriolis forces on micro-rotation profile: the case of linear and quadratic convection.
(3) To observe the variations in skin friction coefficients and Nusselt number against growing values of rotating, magnetic, thermal buoyancy, and material parameters in the case of linear and quadratic convection.

## 2. Mathematical Formulation

**Figure 1:**
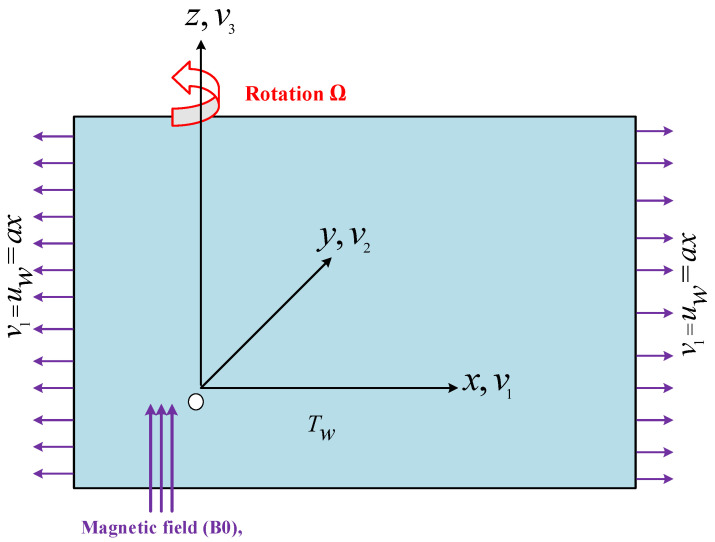
The flow geometry representing the dynamics of rotating micropolar fluid over a stretched surface.

Let us analyze the steady 3D rotational flow of micropolar fluid on a stretching surface in the presence of magnetic effects subject to linear and nonlinear convection. Two forces act in opposite directions to each other and identically act in the direction of *x*-axis to stretch the sheet at a velocity of $u_{w}=ax$, and a>0 is the stretching rate. $B_{o}$ denotes the static magnetic effect which is uniformly acting in the *z*-direction, and under the small magnetic field Reynolds number assumption, the induced magnetic field is negligible. Further, Ohmic dissipation and Hall current impacts are neglected because the applied magnetic field is not very strong [23,24]. The viscous dissipation term is commonly discussed in very high-speed turbulent fluid flow models, particularly in situations where heat transfer is critical. In the present elaborated fluid model, the fluid speed is not fast, and fluid motion generates kinetic energy, which changes the internal fluid energy. This is an irreversible mechanism that causes localized fluid heating. The viscous dissipation term in the energy equation is usually insignificant compared to the convective and diffusive factors. That’s why viscous dissipation is ignored in this model [25,26]. Furthermore, isothermal conditions are sustained at the sheet surface, and viscous dissipation is neglected. Moreover, it is considered that $T_{\infty }$ and $T_{w}$ are ambient temperature and temperature at the surface. The elaborated fluid flow problem geometry is depicted in Figure 1. Because of these assumptions, the boundary layer equations governing the conservation of mass, linear momentum, micro-rotation, and thermal energy in vector form are as follows [27,28,29]:

(1) $$\nabla \cdot (\rho \vec{V})=0,$$

(2) $$\begin{array}{c} \left[\left(\vec{V}\cdot \nabla \right)\vec{V}+\left(\vec{\Omega }\times \left(\vec{\Omega }\times \vec{r}\right)\right)+\left(2\vec{\Omega }\times \vec{V}\right)\right]=-\frac{\nabla p}{\rho }+\left(\nu +\frac{k}{\rho }\right)\nabla^{2}\vec{V}+\frac{\kappa }{\rho }\left(\nabla \times \vec{N}\right)+{\vec{F}}^{*}+\\ \left[\vec{g}\left(\beta_{o}\right)\left(T-T_{\infty }\right)+\vec{g}\left(\beta_{1}\right){\left(T-T_{\infty }\right)}^{2}\right], \end{array}$$

(3) $$\begin{array}{c} \rho j\left[\left(\vec{V}\cdot \nabla \right)\vec{N}\right]=\gamma \nabla^{2}\vec{N}-2\kappa \vec{N}+\kappa \left(\nabla \times \vec{N}\right), \end{array}$$

(4) $$\begin{array}{c} \left[(\vec{V}\cdot \nabla )T\right]=\alpha \nabla^{2}T. \end{array}$$

Here, $\vec{V}=(v_{1},v_{2},v_{3}$) are velocity components in the (x,y,z) directions, $\beta_{o}$, $\beta_{1}$, $\vec{g}$, *T*, $\vec{N}$, ν, $\alpha ,\;{\vec{F}}^{*}$ are the linear volumetric thermal expansion coefficient, nonlinear volumetric thermal expansion coefficient, acceleration due to gravity, fluid temperature, micro-rotation, kinematic viscosity, heat diffusion, and external body forces, respectively. The quantities *j*, *k*, γ, and ρ are micro-inertia per unit mass, vortex viscosity, spin gradient viscosity, and fluid density, respectively. $\vec{\Omega }=\left(0,0,\Omega \right)$ is the angular velocity vector. The equation $\vec{\Omega }\times \left(\vec{\Omega }\times \vec{r}\right)=-\nabla \left(\nabla^{2}r^{2}2\right)$ is given with respect to the centrifugal force, which is being balanced by the pressure gradient −∇p.

By considering the above assumptions, the governing equations are as follows [30,31]:

(5) $$\begin{array}{c} \frac{\partial v_{1}}{\partial x}+\frac{\partial v_{2}}{\partial y}+\frac{\partial v_{3}}{\partial z}=0, \end{array}$$

(6) $$\begin{array}{c} v_{1}\frac{\partial v_{1}}{\partial x}+v_{2}\frac{\partial v_{1}}{\partial y}+v_{3}\frac{\partial v_{1}}{\partial z}-2\Omega v_{2}=\left(\nu +\frac{k}{\rho }\right)\frac{\partial^{2}v_{1}}{\partial z^{2}}+\frac{k}{\rho }\frac{\partial N}{\partial z}-\frac{\sigma }{\rho }B_{o}^{2}v_{1}+\left[g\left(\beta_{o}\right)\left(T-T_{\infty }\right)+g\left(\beta_{1}\right){\left(T-T_{\infty }\right)}^{2}\right], \end{array}$$

(7) $$\begin{array}{c} v_{1}\frac{\partial v_{2}}{\partial x}+v_{2}\frac{\partial v_{2}}{\partial y}+v_{3}\frac{\partial v_{2}}{\partial z}+2\Omega v_{1}=\left(\nu +\frac{k}{\rho }\right)\frac{\partial^{2}v_{2}}{\partial z^{2}}-\frac{\sigma }{\rho }B_{o}^{2}v_{2}, \end{array}$$

(8) $$\begin{array}{c} v_{1}\frac{\partial N}{\partial x}+v_{2}\frac{\partial N}{\partial y}+v_{3}\frac{\partial N}{\partial z}=\frac{\gamma }{\rho j}\frac{\partial^{2}N}{\partial z^{2}}-\frac{k}{\rho j}\left(2N+\frac{\partial v_{1}}{\partial z}\right), \end{array}$$

(9) $$\begin{array}{c} v_{1}\frac{\partial T}{\partial x}+v_{3}\frac{\partial T}{\partial z}+v_{2}\frac{\partial T}{\partial y}=\alpha \frac{\partial^{2}T}{\partial z^{2}}. \end{array}$$

The boundary conditions are

(10) $$\begin{array}{c} \left.\begin{array}{c} v_{1}=ax,\;v_{2}=0,\;v_{3}=0,\;N=-\beta \frac{\partial v_{1}}{\partial z},\;T=T_{w},\;at\;z=0,\\ v_{1}⟶0,\;v_{2}⟶0,\;N⟶0,\;T⟶T_{\infty },\;when\;z⟶\infty . \end{array}\right\} \end{array}$$

In Equations (8) and (10), the spin gradient viscosity (γ) can be written as [32,33]: γ=(μ+κ2)j, where $j=\nu ax^{0.5}$, and the β is a boundary constant parameter with 0≤β≤1. The similarity transformation that has been used to in the analysis is the following [30,34]:

(11) $$\begin{array}{c} \eta =\sqrt{\frac{a}{v}}z,\;v_{1}=axF_{1}^{\prime }\left(\eta \right),\;v_{3}=-{\left(a\nu \right)}^{\frac{1}{2}}F_{1}\left(\eta \right),\;v_{2}=axF_{2}\left(\eta \right),\;N=ax\sqrt{\frac{a}{\nu }}H\left(\eta \right),\;\Theta \left(\eta \right)=\frac{T-T_{\infty }}{T_{w}-T_{\infty }}. \end{array}$$

By use of similarity transformation, Equation (5) is satisfied identically. By using Equation (11), the Equations (6)–(9) are converted into following ODEs:

(12) $$\begin{array}{c} \left(1+K\right)F_{1}^{\prime \prime \prime }+F_{1}F_{1}^{\prime \prime }+2\Gamma F_{2}+KH^{\prime }-F_{1}^{\prime 2}-MF_{1}^{\prime }+\lambda_{1}\left[\theta +\lambda_{2}{\left(\theta \right)}^{2}\right]=0, \end{array}$$

(13) $$\begin{array}{c} \left(1+K\right)F_{2}^{\prime \prime }-F_{1}^{\prime }F_{2}+F_{1}F_{2}^{\prime }-2\Gamma F_{1}^{\prime }-MF_{2}=0, \end{array}$$

(14) $$\begin{array}{c} \left(1+\frac{K}{2}\right)H^{\prime \prime }+F_{1}H^{\prime }-F_{1}^{\prime }H-K\left(2H+F_{1}^{\prime \prime }\right)=0, \end{array}$$

(15) $$\begin{array}{c} \Theta^{\prime \prime }+Prf\Theta^{\prime }=0, \end{array}$$

and the boundary conditions are:

(16) $$\begin{array}{c} \left.\begin{array}{c} F_{1}^{\prime }\left(0\right)=1,\;F_{1}\left(0\right)=0,\;F_{2}\left(0\right)=0,\;H=-\beta F_{1}^{\prime \prime },\;\Theta \left(0\right)=1,\;at\;\eta =0,\\ F_{1}^{\prime }\left(\infty \right)⟶0,\;F_{2}\left(\infty \right)⟶0,\;H=0,\;\Theta \left(\infty \right)⟶0,\;at\;\eta ⟶0. \end{array}\right\} \end{array}$$

In Equations (9)–(12), $\lambda_{1}$ is mixed convection parameter, *K* is the material parameter, $\lambda_{2}$ is the nonlinear or quadratic convection parameter, H is micro rotation, the rotational parameter is Γ, M and Pr are magnetic parameter and Prandtl number, respectively, which are defined as:

(17) $$\begin{array}{c} \lambda_{1}=\frac{Gr_{x}}{Re_{x}^{2}},\;\lambda_{2}=\left(\frac{\beta_{1}}{\beta_{o}}\right)\left(T_{w}-T_{\infty }\right),\\ K=\frac{k}{\mu },\;\Gamma =\frac{\Omega }{a},\;M=\frac{\sigma_{nf}B_{o}^{2}}{\rho_{nf}a},\;Pr=\frac{{\left(\mu C_{p}\right)}_{f}}{k_{f}}, \end{array}$$

$Gr_{x}=\frac{g{\left(\beta_{o}\right)}_{bf}\left(T_{w}-T_{\infty }\right)x^{3}}{v_{bf}^{2}}$ is Local Grashof number and $Re_{x}=\frac{ax^{2}}{v_{bf}}$ is Reynolds number.

Relations for coefficient of skin friction and Nusselt number are defined as:

(18) $$\begin{array}{c} \left.\begin{array}{c} Cf_{x}=\frac{\tau_{w}^{x}}{\rho {\left(ax\right)}^{2}},\\ Cf_{y}=\frac{\tau_{w}^{y}}{\rho {\left(ax\right)}^{2}},\\ Nu_{x}=\frac{xq_{w}}{k_{f}\left(T-T_{\infty }\right)}, \end{array}\right\} \end{array}$$

here, the tensor of skin friction at the wall are $\tau_{w}^{x}=\left[\kappa N+\left(\mu +\kappa \right){\left(\partial v_{1}\partial z\right)}_{z=0}\right]$ (x-direction) and $\tau_{w}^{y}=\left[\kappa N+\left(\mu +\kappa \right){\left(\partial v_{2}\partial z\right)}_{z=0}\right]$ (y-direction), and heat flux at the sheet are $q_{w}=-\kappa {\left(\partial T/\partial z\right)}_{z=0}$. Using the similarity transformation Equation (11), expression described in Equation (18) give the following skin friction and Nusselt number as:

(19) $$\begin{array}{c} \left.\begin{array}{c} {\left(Re_{x}\right)}^{0.5}Cf_{x}=\left(1+\left(1-\beta \right)K\right)F_{1}^{\prime \prime }\left(0\right)\\ {\left(Re_{x}\right)}^{0.5}Cf_{y}=\left(1+\left(1-\beta \right)K\right)F_{2}^{\prime }\left(0\right)\\ {\left(Re_{x}\right)}^{-0.5}Nu_{x}=-\Theta^{\prime }\left(0\right), \end{array}\right\} \end{array}$$

where $Re_{x}=ax^{2}\nu$ is the local Reynolds number.

## 3. Solution Procedure

In this section, the ordinary nonlinear coupled differential flow expressions (12)–(15), subject to conditions in (16), are addressed and solved numerically by utilizing the computational tool bvp4c, a built-in function in MATLAB software. The nonlinear system of coupled flow equations is changed to first-order differential equations as following the references [35,36]:

$D_{1}^{\prime }=D_{2},$
$D_{2}^{\prime }=D_{3},$
$D_{3}^{\prime }=\frac{\left(-1\right)}{\left(1+K\right)}\left[D_{1}D_{3}+2\Gamma D_{4}-KD_{7}-D_{2}^{2}-MD_{2}+\lambda_{1}\left(D_{8}+\lambda_{2}D_{8}^{2}\right)\right],$
$D_{4}^{\prime }=D_{5},$
$D_{5}^{\prime }=\frac{\left(-1\right)}{\left(1+K\right)}\left[D_{1}D_{5}-D_{2}D_{4}-2\Gamma D_{2}-MD_{4}\right],$
$D_{6}^{\prime }=D_{7},$
$D_{7}^{\prime }=\frac{\left(-1\right)}{\left(1+\frac{K}{2}\right)}\left[D_{1}D_{7}-K\left(2D_{6}-D_{3}\right)\right],$
$D_{8}^{\prime }=D_{9}$
$D_{9}^{\prime }=-PrD_{1}D_{9},$

The corresponding boundary conditions are as follows:

$$\begin{array}{c} D_{1}=0,\;D_{2}=1,\;D_{4}=0,\;D_{7}=-\beta D_{3},\;D_{9}=1,\;at\;\eta =0,\\ D_{2}\to 0,\;D_{4}\to 0,\;D_{7}\to 0,\;D_{9}\to 0,\;as\;\eta \to \infty . \end{array}$$

The above first-order differential equations with seven initial conditions are solved via the shooting technique. The numerical computation has been performed for various physical emerging parameters for the appropriate computational domain [0,10] instead of [0,*∞*], where η is fixed at 10 because there is no more variation in the results after η=10. Newton’s iterative scheme is applied to improve the accuracy of the initial guesses until the desired approximation is obtained. The stopping criteria for the iterative process is $10^{-6}$. Bvp4c is an effective solver for a system of ODEs compared to other boundary value problem solvers. It is elementary to implement on MATLAB and has a low computational cost.

## 4. Results and Discussion

**Figure 2:**
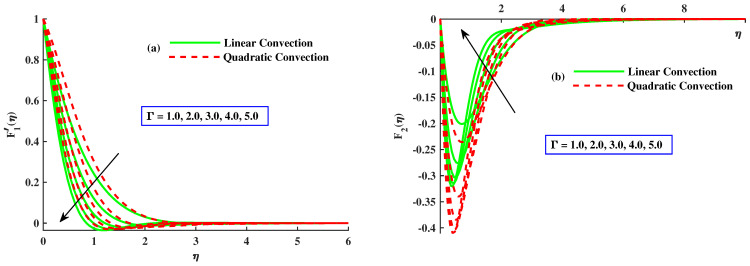
The influence of Γ on the ${F_{1}}^{\prime }\left(\eta \right)$ and $F_{2}\left(\eta \right)$.

**Figure 3:**
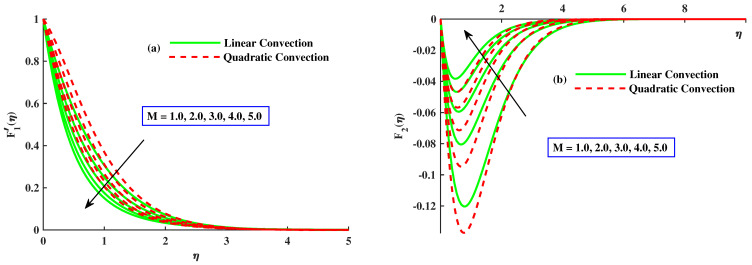
The influence of *M* on the ${F_{1}}^{\prime }\left(\eta \right)$ and $F_{2}\left(\eta \right)$.

**Figure 4:**
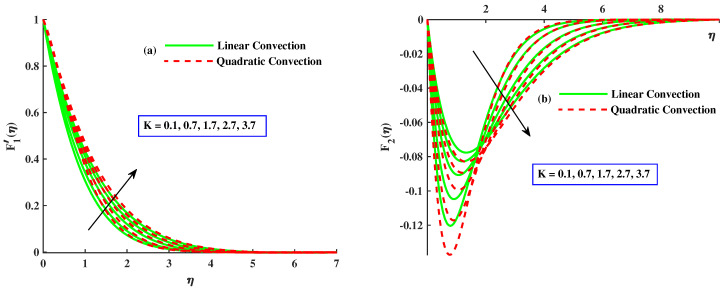
The influence of *K* on the ${F_{1}}^{\prime }\left(\eta \right)$ and $F_{2}\left(\eta \right)$.

**Figure 5:**
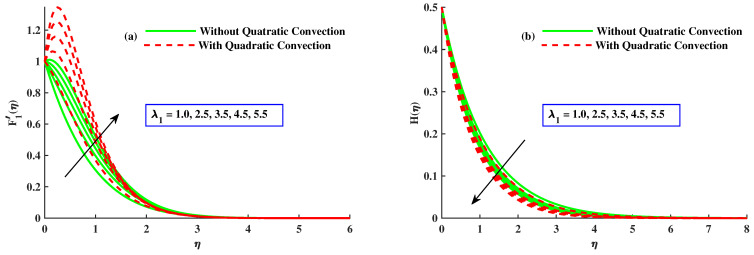
The influence of $\lambda_{1}$ on the ${F_{1}}^{\prime }\left(\eta \right)$ and H(η).

**Figure 6:**
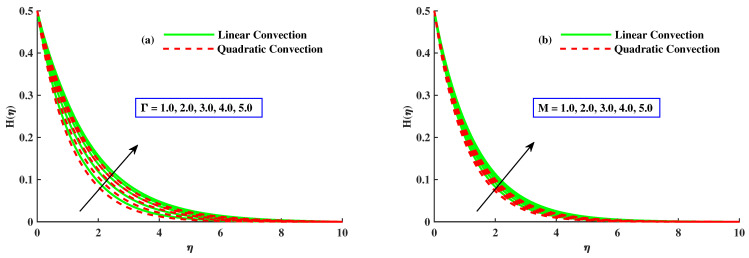
The influence of Γ&M on the H(η).

**Figure 7:**
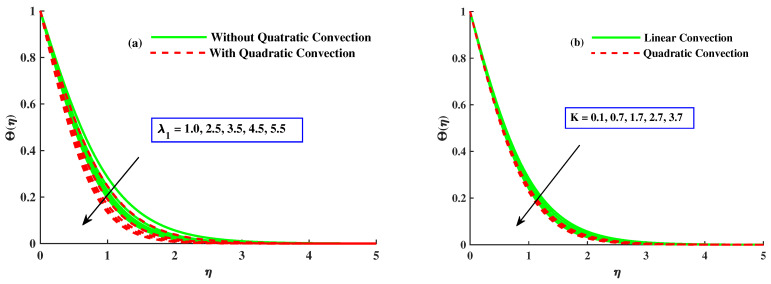
The influence of $K\;\&\;\lambda_{1}$ on the Θ(η).

**Figure 8:**
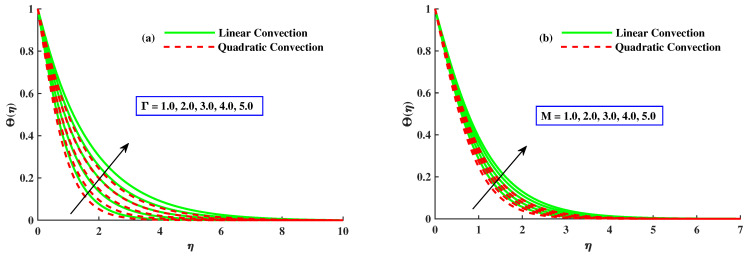
The influence of Γ&M on the Θ(η).

**Figure 9:**
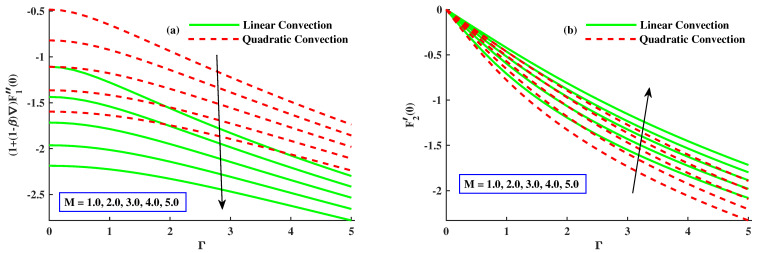
The influence of Γ&M on the $Cf_{x}{\left(Re\right)}^{0.5}$ and $Cf_{y}{\left(Re\right)}^{0.5}$.

**Figure 10:**
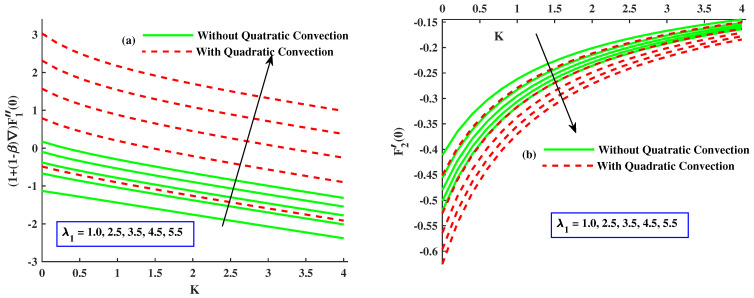
The influence of $\lambda_{1}\;\&\;K$ on the $Cf_{x}{\left(Re\right)}^{0.5}$ and $Cf_{y}{\left(Re\right)}^{0.5}$.

**Figure 11:**
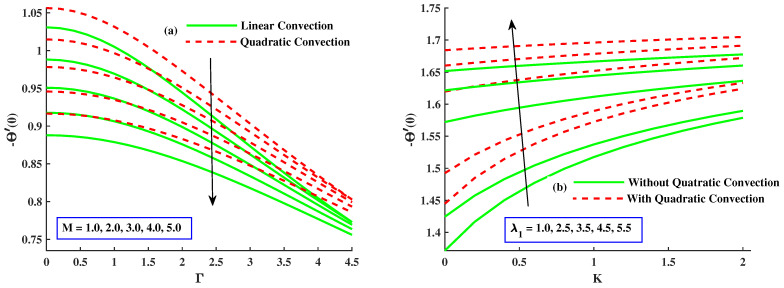
The influence of $M,\;\Gamma ,\;\;K,\;\&\;\lambda_{1}$ on the $Nu_{x}Re_{x}^{0.5}$.

This section emphasizes the physical parameters’ significance on velocity, micro-rotation, and temperature profile, and the behavior of Nusselt number and skin friction coefficients for different values of involved parameters. The results comparison is conducted for limited cases to verify the present results as presented in Table 1 and Table 2, and an excellent relation between already existing results is found. For the factor of skin friction, a comparison of results with Wang et al. [18] and Ali et al. [37] for various values of rotating parameters when other parameters are ignored, as presented in Table 1. The consequences for Nusselt number against rotating parameter and Pr are compared with Awan et al. [38] and Ali et al. [39] in Table 2. An excellent correlation has been achieved, which affirms the validity of bvp4c utilization. Estimation of the current analysis is done by using the following values of parameters: Γ=0,5, Pr=3, $\lambda_{1}=1.0$, M=1.0, $\lambda_{2}=2.0$, K=0.1 and β=0.5. Figure 2, Figure 3, Figure 4, Figure 5, Figure 6, Figure 7, Figure 8, Figure 9, Figure 10, and Figure 11, are plotted for two cases, namely:

Case 1: with linear convection ($\lambda_{1}=1.0$, $\lambda_{2}=0.0$).
Case 2: with quadratic convection ($\lambda_{1}=1.0$, $\lambda_{2}=2.0$).

Figure 2a,b demonstrates the influence of rotating parameter with linear and quadratic convection on the velocity profile. It demonstrates that, by enhancement of rthe otating parameter, the primary velocity $F_{1}^{\prime }$ and magnitude of secondary velocity $F_{2}$ decrease and the linear convection have same behavior for velocity profile but, with quadratic convection, the velocity $F_{1}^{\prime }$ increases, and velocity $F_{2}$ reduces. Dual behavior is detected in the distribution of the fluid velocity. Far from the body surface, the increment in quadratic convection has diminished the velocity, but a reverse tendency is noted in the zone close to the body surface. Physically, the nonlinear thermal parameter is directly proportional to the temperature gradient of the surface, and buoyancy force is produced consequently to boost the velocity profile. Figure 3a,b reveals the effect of magnetic parameters with linear and nonlinear convection on velocities $F_{1}^{\prime }$ and $F_{2}$. It is clear that both velocities are decreased by enlarging the value of the magnetic parameter. Physically, growing values of *M* means an increase in drag force, that is, the force which slows the velocity. The velocity profile decreased against the linear convection case and the secondary velocity has increasing behavior against quadratic convection. The effect of material parameters on velocity profile is sketched out in Figure 4a,b. By incrementing values of *K* with quadratic convection, $F_{1}^{\prime }$ amplified but $F_{2}$ reduced, and in the presence of linear convection, the velocities lessened. Physically, the material parameter is inversely proportional to coefficients of dynamic viscosity; therefore, its higher values allow the fluid flow to move fast.

The influence of mixed convection parameter $\lambda_{1}$ on velocity $F_{1}^{\prime }$ and micro rotation distribution profiles, with and without quadratic convection, is shown in Figure 5a,b. The velocity increased with higher inputs of mixed convection parameter and has rising behavior for quadratic convection case but is reduced without quadratic convection as depicted in Figure 5a. Figure 5b displays the effect of $\lambda_{1}$ on the micro-rotation distribution profile. With increased values of mixed convection parameter, H(η) decreases and behaves as for quadratic convection but increases without the quadratic convection case. Figure 6a,b portrays the effect of rotating and magnetic parameters on micro-rotation in the linear and quadratic convection cases. Figure 6a discloses that with increased values of the rotating parameter, H(η) increases, and it also increases with linear convection, but it has the opposite behavior against quadratic convection. The increases in micro-rotation with rising inputs of the magnetic parameter along linear convection, and the reverse trend with quadratic convection, can be seen in Figure 6b.

Figure 7a illustrates the effect of mixed convection parameter along and without quadratic convection on the temperature distribution. The figure reveals that the temperature profile decreased with more significant inputs of $\lambda_{1}$ with quadratic convection and an evident increase without quadratic convection. Figure 7b explains the effect of variation of the material parameter with both cases on θ. Temperature profile is reduced by increasing values of *K* with nonlinear convection but has a rising tendency along linear convection. Figure 8a,b presents the effect of magnetic and rotating parameters on the temperature of the fluid. The fluid’s temperature rises with variational values of the rotating parameter and thermal convection. However, it reduces with quadratic convection, as indicated in Figure 8a. Physically, heat development justification is based on the raised diffusion process due to higher inputs Γ. Figure 8b represents that the temperature of the liquid is boosted with increasing values of the magnetic parameter with linear convection but lessened with nonlinear convection. Fluid flux is stopped, and dissipation appends to the fluid’s thermal energy with magnified values of the magnetic parameter, so the temperature rises.

Figure 9a,b are drawn to show the variation in $Cf_{x}{\left(Re\right)}^{0.5}$ and $Cf_{y}{\left(Re\right)}^{0.5}$ when magnetic and quadratic parameters are given distinct values with linear and nonlinear convection. The coefficient of skin friction ($Cf_{x}{\left(Re\right)}^{0.5}$) decreased when *M*, *K* enhanced and also in the case of linear convection, but it has amplifying behavior along with quadratic convection, and the coefficient of skin friction ($Cf_{y}{\left(Re\right)}^{0.5}$) undergoes a notable increment by higher values of *M* along linear convection, but it shows a significant decrement by greater inputs of *K* with quadratic convection. Figure 10a,b indicates the fluctuation in $Cf_{x}{\left(Re\right)}^{0.5}$ and $Cf_{y}{\left(Re\right)}^{0.5}$ with varying values of material and mixed convection parameter along quadratic and without nonlinear convection. Figure 10a represents the increment in $Cf_{x}{\left(Re\right)}^{0.5}$ with growing inputs of λ and, in the case of nonlinear convection. However, it recedes with incremental values of Γ and in the absence of nonlinear convection. Figure 10b displays that $Cf_{y}{\left(Re\right)}^{0.5}$ increased with larger values of the material parameter with linear convection and decreased by varying values of $\lambda_{1}$ with quadratic convection. Figure 11a,b discloses that Nusselt number ($Nu{\left(Re\right)}^{0.5}$) is lessened with elevated inputs of *M* and Γ in both cases but boosted with increasing values of $\lambda_{1}$ and *K* with quadratic convection.

## 5. Conclusions

The linear and quadratic convection flux of non-Newtonian fluid in 3D on extending surfaces in the presence of magnetohydrodynamics is investigated in this study. Fluid velocity, micro-rotation, temperature distribution, skin friction factor, and Nusselt number are conducted using the technique of bvp4c. A few significant consequences are summarized as follows:

- The primary ($F_{1}^{\prime }\left(\eta \right)$) and secondary ($F_{2}\left(\eta \right)$) velocities significantly decrease along with rising values of magnetic (*M*) parameter, rotating (Γ) parameter, and in case of linear convection. However, $F_{1}^{\prime }$ has a growing tendency for the material parameter (*K*), thermal buoyancy ($\lambda_{1}$), and quadratic convection case. The magnitude of $F_{2}$ has a decreasing behavior for nonlinear convection and material parameter.
- The incremented inputs of rotating, magnetic, and the case of without quadratic convection are augmented the micro-rotation distribution. However, for thermal buoyancy and nonlinear convection case, the micro-rotation profile (H(η)) decreases.
- The fluid particles’ temperature increases with higher inputs of rotating and magnetic parameters in linear convection case but is reduced against growing strength of rotating ($\lambda_{1}$) and material (*K*) parameters in quadratic convection case.
- Along the *x* direction, the skin friction coefficient decreases with the amplified value of magnetic, rotating, and material parameters in the linear convection case. Along the *y* direction, the skin friction coefficient is raised by enlargement in magnetic and material parameters without quadratic convection case. However, it decreases for thermal buoyancy and rotating parameters in the quadratic convection case.
- The magnitude of the Nusselt number is reduced with a higher contribution of rotating and magnetic parameters in the linear convection case. However, it increases against material and thermal buoyancy in the nonlinear convection case.

## Figures and Tables

**Table 1:** Comparison of $F_{1}^{\prime \prime }\left(0\right)$ and $F_{2}^{\prime }\left(0\right)$ values along variational inputs of Γ when ignoring other involved parameters.

Γ	Wang et al. [18]		Ali et al. [37]		Current Results	
	$-F_{1}^{\prime \prime }\left(0\right)$	$-F_{2}^{\prime }\left(0\right)$	$-F_{1}^{\prime \prime }\left(0\right)$	$-F_{2}^{\prime }\left(0\right)$	$-F_{1}^{\prime \prime }\left(0\right)$	$-F_{2}^{\prime }\left(0\right)$
0	1.00	0.00	1.000	0.000	1.0000	0.0000
1	1.32	0.83	1.325	0.837	1.3251	0.8372
2	1.65	1.28	1.652	1.287	1.6524	1.2874

**Table 2:** Comparison values of $\Theta^{\prime }\left(0\right)$ with various values of Γ and Pr.

Γ	Ali et al. [39]		Aziz Ullah et al. [38]		Current Results	
	Pr=2.0	Pr=7.0	Pr=2.0	Pr=7.0	Pr=2.0	Pr=7.0
0	0.911	1.895	0.9114	1.8955	0.91135	1.89541
0.5	0.853	1.851	0.8535	1.8512	0.85344	1.85018
1	0.771	1.787	0.6383	1.7877	0.77034	1.787625

## Data Availability

Not applicable.

## Nomenclature

*T*	non-dimensional temperature, K
$T_{\infty }$	temperature away from the surface, K
$Nu_{x}$	Nusselt number
$Cf_{x}$	skin friction at x-direction
$Cf_{y}$	skin friction at y-direction
ν	kinematic viscosity of nanofluid, m${}^{-2}$s${}^{-1}$
Cp	specific heat at constant pressure, JKg${}^{-1}$K${}^{-1}$
σ	electrical conductivity of fluid, Kg${}^{-1}$m${}^{3}$A${}^{2}$
Pr	Prandtl number
μ	dynamic viscosity of fluid, Kgm${}^{-1}$s${}^{-1}$
$q_{w}$	surface heat flux, Wm${}^{-2}$
α	thermal conductivity of the fluid, Wm${}^{-1}$K${}^{-1}$
$T_{w}$	temperature at surface, K
$B_{0}$	Uniform magnetic field
Ω	angular velocity, s${}^{-1}$
$\left(v_{1},v_{2},v_{3}\right)$	Velocity components
$u_{w}$	velocity of stretching sheet, ms${}^{-1}$
ρ	Density of nanofluid, Kgm${}^{-3}$
$B_{0}$	Uniform magnetic field
*K*	Material parameter
(x,y,z)	Cartesian co-ordinates
Γ	rotation parameter
η	dimensionless variable
*M*	magnetic parameter
